# Outcomes of Urgent Transcatheter Aortic Valve Replacement in Patients With Acute Decompensated Heart Failure: A Single-Center Experience

**DOI:** 10.7759/cureus.10425

**Published:** 2020-09-13

**Authors:** Kai Chen, Kayla Polcari, Taylor Michiko, Jesus E Pino, Mark Rothenberg, Cristiano Faber, Marcos Nores, Sotiris Stamou, Waqa Ghumman, Robert Chait

**Affiliations:** 1 Internal Medicine, University of Miami/John F. Kennedy (JFK) Medical Center, Atlantis, USA; 2 Internal Medicine, University of Miami Miller School of Medicine, Atlantis, USA; 3 Cardiovascular Medicine, University of Miami/John F. Kennedy (JFK) Medical Center, Atlantis, USA; 4 Cardiothoracic Surgery, John F. Kennedy (JFK) Medical Center, Atlantis, USA

**Keywords:** tavr, urgent, acute decompensated heart failure

## Abstract

Background

Data on urgent transcatheter aortic valve replacement (TAVR) as rescue therapy for acute decompensated heart failure (ADHF) due to severe aortic stenosis (AS) are limited. We sought to investigate the outcomes of patients who underwent urgent transcatheter aortic valve replacement (TAVR) in a single institution.

Methods

This is a retrospective cohort study of 602 patients with a history of heart failure (HF) due to AS who underwent TAVR between April 2012 and July 2017. We stratified patient cohort into urgent (n=139) and elective (n=463) TAVR. Urgent TAVR was defined as patients who were admitted for ADHF and underwent TAVR during the same hospitalization. Patients that underwent urgent TAVR for other reasons were excluded.

Results

Rates of postoperative intra-aortic balloon pump requirement, atrial fibrillation, dialysis requirement, vascular complications, and stroke were similar between the two groups. Compared with elective TAVR, patients undergoing urgent TAVR had a higher rate of cardiac arrest (5.7% vs 1.3%, p=0.005), longer length of stay (LOS) (11 vs. 5, p<0.001), and significant 30-day mortality (8.6% vs 4.1%, HR 2.1, 95% CI 1.04-4.22). Patients who underwent urgent TAVR were also associated with long-term mortality (Log-rank p = 0.0162).

Conclusions

In our study, urgent TAVR for ADHF was associated with both short-term and long-term mortality as compared to elective TAVR. Further randomized studies are needed to investigate the appropriate management of this population.

## Introduction

Aortic stenosis (AS) is the most common valvular heart disease in the Western world, and nearly 3.4% of the elderly population has severe AS [1]. Symptomatic severe aortic stenosis (SAS) is associated with reduced survival without surgery. If left untreated, more than half of the patients will die at two years [2]. Patients with a history of SAS and heart failure may experience acute decompensation, which requires urgent intervention. Balloon aortic valvuloplasty (BAV) was considered emergent “bridging” therapy for these patients, however, it has been shown to be associated with frequent complications such as only short-term efficacy and the risk of postoperative severe aortic regurgitation [3]. Previously, transcatheter aortic valve replacement (TAVR) had emerged as an effective therapy for patients at prohibitive to low surgical risks [4-6]. Recent studies have found that urgent or emergent TAVR might be feasible and effective in patients with severe AS and acute decompensation [7-9].

However, the outcomes of urgent TAVR remain unclear, with some studies endorsing promising clinical outcomes and others demonstrating increased morbidity and mortality [7-10]. Furthermore, there is concern that patients requiring urgent TAVR may have a limited or absent preoperative evaluation, which could negatively impact the safety and efficacy of the procedure [8,11].

Since unstable patients requiring urgent intervention were excluded from previous randomized clinical trials, data on urgent TAVR for acute decompensated heart failure (ADHF) in patients with SAS is limited and conflicting. Our aim was to investigate the outcomes of patients who underwent urgent TAVR as compared to those who did so electively in a single institution.

## Materials and methods

Study design

This is a retrospective cohort study of prospectively collected data from consecutive patients who underwent TAVR at JFK Medical Center between August 2018 and January 2019. All TAVR patients who had a history of heart failure due to aortic stenosis were identified. They were stratified into two cohorts, including urgent TAVR (n=139) and elective TAVR (n=463). Their preoperative characteristics, intraoperative variables, and postoperative outcomes were collected and compared. Study approval was sought and obtained from the institutional review board. Patient confidentiality was maintained at all times, consistent with the Health Insurance Portability and Accountability Act of 1996 (HIPAA) regulations.

Definitions

Urgent TAVR included those patients who underwent TAVR during the same admission for symptoms of acutely decompensated heart failure. Acute decompensated heart failure is defined by the need for intravenous medical therapy, including diuretics and inotropic drugs. Elective TAVR included those patients admitted electively for planned TAVR. Variables were defined according to the Society of Thoracic Surgeons’ (STS) national cardiac surgery database. Diabetes included those with a history of type 1 or 2 diabetes mellitus, regardless of disease duration or insulin requirement. Prolonged ventilator requirement involved pulmonary insufficiency requiring greater than 24 hours of ventilatory support. Operative mortality included all deaths during the index hospitalization, regardless of cause or timing, and all deaths occurring within 30 days of the operation [12].

Data analysis

Categorical variables were summarized using frequencies and percentages while continuous variables were reported using the median and interquartile range. Comparisons of preoperative, operative, and postoperative characteristics were performed between the patients who underwent an urgent versus elective TAVR using Pearson’s chi-squared or Fisher’s exact test where statistically appropriate. Continuous data were compared using the Wilcoxon rank-sum test. The Kaplan-Meier method was used to determine the 30-day and five-year survival of patients in the two cohorts. All tests were two-sided, where applicable, with a p-value of <0.05 considered statistically significant. All statistical analyses were performed using SAS v9.4 statistical software for Windows (SAS Institute Inc., Cary, NC).

## Results

Preoperative characteristics

Patients who underwent elective versus urgent TAVR were largely similar in preoperative characteristics (Table 1). Demographics, including age, sex, and race, were comparable between groups, with approximately 58% males in both groups at a median age of 86. Patients also had similar rates of comorbidities, including atrial fibrillation, hypertension, diabetes, tobacco use, chronic obstructive pulmonary disease (COPD), and peripheral vascular disease. There was no significant difference in the prior history of myocardial infarction (MI), stroke, aortic valve replacement, or coronary artery bypass graft (CABG). Aortic valve parameters, such as aortic valve area, velocity, and pressure gradient, were also comparable between groups. Compared to patients admitted electively for TAVR, those who underwent the procedure urgently had significantly higher STS risk scores (6.8 vs. 5.17, p<0.0001), reduced ejection fractions (57 vs. 50, p<0.0001), and more severe New York Heart Association (NYHA) class (p=0.0032), consistent with acute heart failure decompensation. Three-quarters of patients in both groups had NYHA class III heart failure at the time of TAVR, however, more patients in the urgent group had class IV heart failure (5.40% in the elective group vs. 11.51% in the urgent group). 

**Table 1:** Preoperative characteristics Variables are expressed as numbers (%) or median (interquartile range) TAVR = Transcatheter aortic valve replacement, STS = Society of Thoracic Surgery, COPD = Chronic obstructive pulmonary disease, ΔPmean = Mean gradient, AVA = Aortic valve area, Vmax = Peak velocity, CABG = Coronary-artery bypass

Baseline Characteristics	Elective TAVR N=463	Urgent TAVR N=139	P-value
Age in years*	86 (81-89)	86 (80-89)	P=0.6709
Female sex, n (%)	192 (41.47%)	59 (42.45%)	P=0.8450
Race or ethnic group	-	-	-
Hispanic	36 (7.78%)	15 (10.79%)	P=0.2949
Black	8 (1.73%)	1 (0.72%)	P=0.6920
White	442 (95.46%)	128 (92.09%)	P=0.2503
STS Risk Score*	5.17 (3.59-7.40)	6.8 (4.9-9.2)	P <0.0001
BMI*	26.09 (23.43-30.26)	26.40 (23.18-30.11)	P=0.6938
Pre-procedure AVA cm^2^*	0.6 (0.5-0.8)	0.6 (0.5-0.8)	P=0.4124
Pre-procedure ΔPmean*	44 (37-52)	46 (38-55)	P=0.0945
Pre-procedure Vmax*	4.1 (3.8-4.6)	4.1 (3.8-4.5)	P=0.8772
Ejection Fraction*	57 (45-65)	50 (40-60)	P <0.0001
Atrial Fibrillation	218 (47.08%)	66 (47.48%)	P=1.0000
Current smoker	14 (3.02%)	5 (3.60%)	P=0.7810
Hypertension	414 (89.42%)	128 (92.09%)	P=0.4214
COPD	174 (37.58%)	50 (35.97%)	P=0.4917
Diabetes	158 (34.13%)	54 (38.85%)	P=0.3128
NYHA Class			P=0.0032
NYHA class I		1 (0.72%)	-
NYHA class II	71 (15.33%)	11 (7.91%)	-
NYHA class III	351 (75.81%)	104(74.82%)	-
NYHA class IV	25 (5.40%)	16 (11.51%)	-
Previous aortic valve	66 (14.25%)	15 (10.79%)	P=0.3243
Previous CABG	109 (23.54%)	37 (26.62%)	P=0.4992
Peripheral vascular disease	120 (25.92%)	28 (20.14%)	P=0.1790
Previous myocardial infarction	136 (29.37%)	43 (30.94%)	P=0.6701
Previous stroke	46 (9.94%)	17 (12.23%)	P=0.5275

Operative characteristics

Table 2 indicates that operative characteristics were similar between elective and urgent cases, with most patients undergoing TAVR with transfemoral access using Edwards Sapien 3 valve models (Edwards Lifesciences, Irvine, CA). No significant difference in the use of rapid ventricular pacing, BAV, or valve in valve procedures was found between groups. There was, however, a statistically significant difference in the number of balloon post-dilations required in each group (p=0.0013).

**Table 2:** Intraoperative characteristics Variables are expressed as numbers (%) or median (interquartile range) TAVR = Transcatheter aortic valve replacement, BAV = Balloon valvuloplasty

Intraoperative Characteristics	Elective TAVR N=463	Urgent TAVR N=139	P-value
Access Site			P= 0.6273
Transfemoral	374 (80.78%)	115 (82.73%)	
Transapical	47 (10.15%)	13 (9.35%)	
Transaxillary	32 (6.91%)	10 (7.2%)	
Transaortic	7 (1.51%)	6.8 (4.9-9.2)	
Transcarotid	1 (0.22%)	1 (0.72%)	
Rapid ventricular pacing	418 (90.06%)	123 (88.49%)	P=0.8035
Pre BAV	347 (74.95%)	101 (72.7%)	P=0.5774
Post-dilations			P=0.0013
0	301 (65.01%)	110 (79.14%)	
1	143 (30.89%)	21 (15.11%)	
2	16 (3.46%)	6 (4.32%)	
3	2 (0.43%)	.	
Valve size			P=0.2153
20	7 (1.51%)	3 (2.16%)	
23	176 (38.01%)	55 (39.57%)	
26	155 (33.48%)	38 (27.34%)	
29	100 (21.60%)	37 (26.62%)	
31	3 (0.65%)	3 (2.16%)	
34	21 (4.54%)	3 (2.16%)	
Valve model			P=0.8692
Sapien 3	298 (64.36%)	93 (66.91%)	
CoreValve™ Evolut™ R	33 (7.13%)	12 (8.64%)	
Evolut R or Evolut PRO	36 (7.77%)	9 (6.48%)	
Sapien	96 (20.73%)	25 (17.99%)	
Valve in valve	25 (5.40%)	9 (6.47%)	P=0.744

Postoperative outcomes

**Figure 1:**
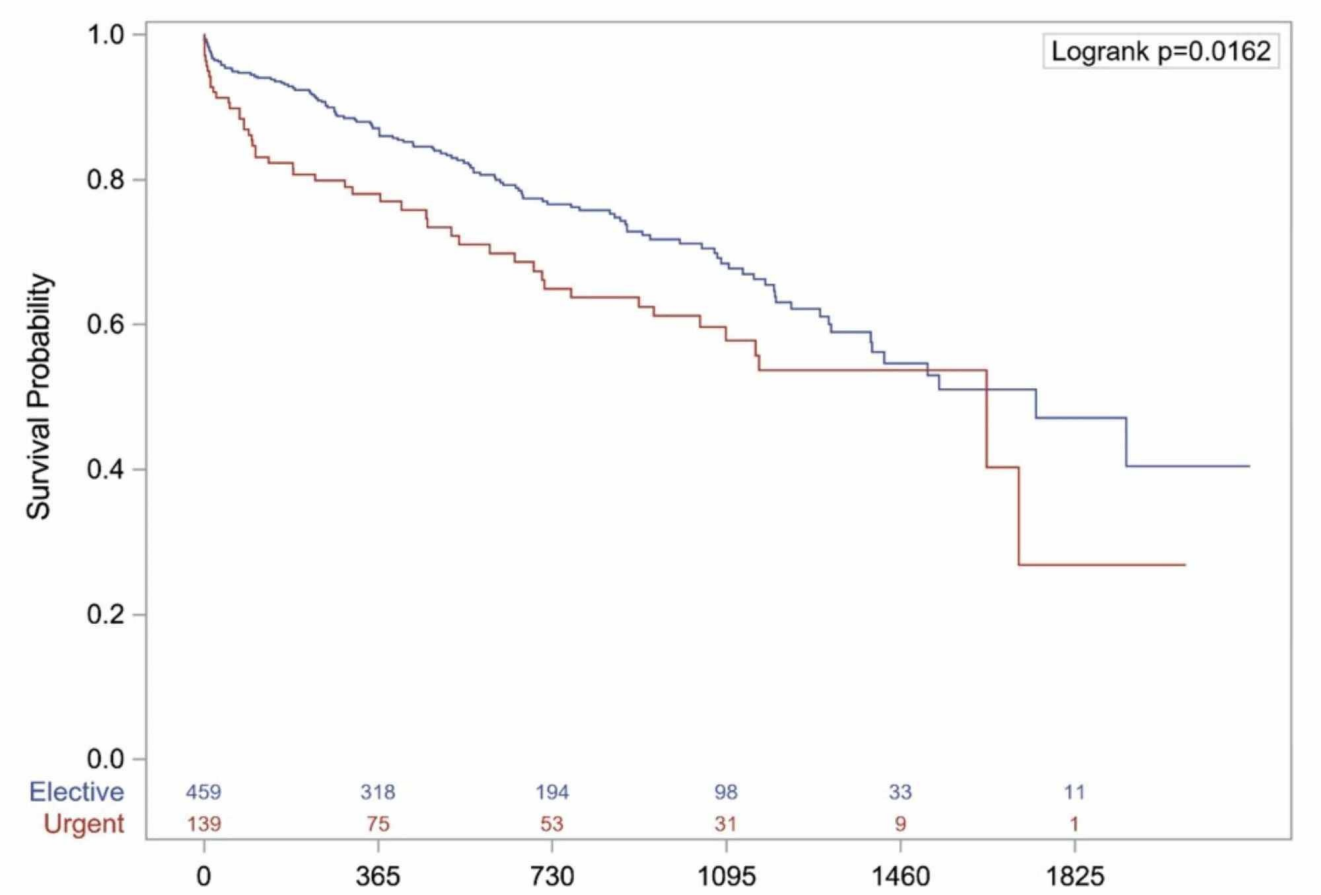
Kaplan-Meier survival analysis Overall five-year Kaplan-Meier survival curve between elective and urgent TAVR. TAVR = Transcatheter aortic valve replacement

Important differences were found in postoperative outcomes between elective versus urgent TAVR patients (Table 3). There were similar rates of new-onset atrial fibrillation, dialysis requirement, intra-aortic balloon pump (IABP) requirement, pacemaker implant, stroke, and vascular complications. Those with urgent TAVR had poorer outcomes, with significantly increased rates of postoperative cardiac arrest (1.30% vs. 5.76%, p=0.0056) and 30-day mortality (4.10% vs. 8.63%, p=0.0468). Although 30-day readmission rates were comparable between groups, those with elective TAVR had significantly greater lengths of stay (5 vs. 11 days, p<0.0001). Further, Kaplan Meier survival analysis (Figure 1) reveals that patients undergoing elective TAVR have a significantly greater probability of five-year survival postoperatively than their acutely decompensated counterparts (p=0.0162).

**Table 3:** Postoperative characteristics Variables are expressed as numbers (%) or median (interquartile range) TAVR = Transcatheter aortic valve replacement, LOS = Length of stay, IABP = Intra-aortic balloon pump

Characteristics	Elective TAVR N=463	Urgent TAVR N=139	P-value
Cardiac arrest	6 (1.30%)	8 (5.76%)	P=0.0056
IABP requirement	2 (0.43%)	1 (0.72%)	P=1.0000
New-onset atrial fibrillation	18 (3.89%)	6 (4.32%)	P=1.0000
New-onset dialysis requirement	9 (1.94%)	2 (1.44%)	P=1.0000
Pacemaker implant	67 (14.47%)	15 (10.79%)	P=0.3239
Postoperative stroke	19 (4.10%)	7 (5.04%)	P=0.8130
Vascular complications	37 (7.99%)	17 (12.23%)	P=0.1298
Prolonged ventilator requirement	19 (4.10%)	9 (6.47%)	P=0.2533
30-day readmission	80 (17.28%)	23 (16.55%)	P=0.8984
30-day mortality	19 (4.10%)	12 (8.63%)	P=0.0468
LOS	5 (3-8)	11 (8-16)	P <0.0001

## Discussion

Principal findings

Compared to elective TAVR patients, those who undergo urgent TAVR have higher STS risk scores, reduced ejection fraction, and more severe NYHA class. This reflects the acutely decompensated nature of their disease at the time of operation, which warrants an urgent procedure within the same hospital admission. Although operative characteristics are similar between groups, patients have worse outcomes in the postoperative period after urgent TAVR, with a significantly greater percentage experiencing cardiac arrest (p=0.0056) and 30-day mortality (p=0.0468). Length of stay (LOS) was also significantly longer for urgent versus elective cases (11 vs. 5 days, p<0.0001). Five-year survival postoperatively is significantly greater in those admitted electively for TAVR than those operated on urgently (log-rank p=0.0162). Thus, urgent TAVR for ADHF is not only associated with increased perioperative mortality but also with increased long-term mortality as compared to elective TAVR. Our findings corroborate those of a recent large registry by Kolte et al. [9], who also noted increased mortality rates at 30 days and one year after urgent or emergent TAVR.

Clinical implications

This study analyzed patients undergoing elective versus urgent TAVR for ADHF. Patients requiring urgent TAVR are sicker, with higher STS risk scores and more severe heart failure, as evidenced by lower ejection fractions and worse NYHA class. Urgent TAVR is associated with worse outcomes, including increased short- and long-term mortality, as is expected in patients with worse baseline clinical status and reduced physiologic reserve [13]. Eliminating the valvular obstruction in AS earlier may improve outcomes in patients with comorbid heart failure. Additionally, by more than doubling the average LOS for TAVR, urgency incurs more costs for both hospitals and patients for a procedure that can already be quite costly [14].

Although there is no clear guideline regarding optimal rescue therapy, BAV had been considered bridge therapy to surgery or TAVR for patients. Bongiovanni et al. reported there was no difference in 30-day mortality between TAVR or BAV in patients who needed urgent intervention due to acute decompensation [3]. Furthermore, in patients with acute cardiogenic shock, BAV was associated with high rates of restenosis and in-hospital mortality of 71% [15]. A recent retrospective study by Frerker et al. concluded TAVR is reasonable rescue therapy in patients with acute cardiogenic shock with a 30-day mortality of 33.3% and there was no significant difference in survival at one-year when compared to the elective cohort [16]. In our study, the 30-day mortality of 8.6% further supports the fact that TAVR should be considered definite therapy in patients with acute decompensation, especially in more experienced centers.

Urgent TAVR and its associated mortality may be avoided by improved identification of symptomatic patients and reduced wait times from referral to the procedure, particularly in patients with worsening heart failure symptoms signaling impending decompensation. Demand for TAVR has been increasing and is expected to continue doing so as indications for TAVR expand [17]. The influx of patients who qualify for TAVR predisposes to longer wait times, placing patients at greater risk for interval decompensation and mortality [18]. Thus, it will be of even greater importance to identify and prioritize those patients at risk for acute decompensation moving forward.

Limitations

Our study was conducted retrospectively at a single institution, predisposing to bias. The sample size was limited, particularly in the urgent TAVR cohort. However, our study indicates that urgent TAVR for ADHF is associated with worse perioperative and long-term outcomes. Further randomized studies are needed to investigate the appropriate management of this population and to establish guidelines on appropriate wait times for TAVR.

## Conclusions

Compared to patients who have elective TAVRs, those undergoing urgent TAVR due to acute decompensation have higher STS risk scores, reduced ejection fraction, and more severe NYHA class preoperatively. Urgent TAVR is associated with worse outcomes as compared to elective TAVR, including increased short- and long-term mortality and longer LOS. These discrepancies are likely attributable to worse baseline clinical status in acutely decompensated patients. A reduction of time from AS diagnosis and symptomatology to valve replacement is indicated to prevent the need for urgent procedures. The need for improved recognition of AS patients at risk for acute decompensation is critical, as urgent TAVR is costly in terms of both lives and resources.

## References

[1] Osnabrugge RL, Mylotte D, Head SJ (2013). Aortic stenosis in the elderly: disease prevalence and number of candidates for transcatheter aortic valve replacement: a meta-analysis and modeling study. J Am Coll Cardiol.

[2] Carabello BA, Green LH, Grossman W, Cohn LH, Koster JK, Collins JJ Jr (1980). Hemodynamic determinants of prognosis of aortic valve replacement in critical aortic stenosis and advanced congestive heart failure. Circulation.

[3] Bongiovanni D, Kuhl C, Bleiziffer S (2018). Emergency treatment of decompensated aortic stenosis. Heart.

[4] Leon MB, Smith CR, Mack M (2010). Transcatheter aortic-valve implantation for aortic stenosis in patients who cannot undergo surgery. N Engl J Med.

[5] Leon MB, Smith CR, Mack MJ (2016). Transcatheter or Surgical Aortic-Valve Replacement in Intermediate-Risk Patients. N Engl J Med.

[6] Mack MJ, Leon MB, Thourani VH (2019). Transcatheter aortic-valve replacement with a balloon-expandable valve in low-risk patients. N Engl J Med.

[7] Abdelaziz M, Khogali S, Cotton JM (2018). Transcatheter aortic valve implantation in decompensated aortic stenosis within the same hospital admission: early clinical experience. Open Heart.

[8] Landes U, Orvin K, Codner P (2016). Urgent transcatheter aortic valve implantation in patients with severe aortic stenosis and acute heart failure: procedural and 30-day outcomes. Can J Cardiol.

[9] Kolte D, Khera S, Vemulapalli S (2018). Outcomes following urgent/emergent transcatheter aortic valve replacement: insights from the STS/ACC TVT registry. JACC Cardiovasc Interv.

[10] Elbaz-Greener G, Yarranton B, Qiu F (2019). Association between wait time for transcatheter aortic valve replacement and early postprocedural outcomes. J Am Heart Assoc.

[11] Kashiyama N, Kuratani T, Torikai K (2015). Urgent transcatheter aortic valve replacement for severe aortic valve stenosis with acute decompensated heart failure: report of a case. Surg Today.

[12] Overman DM, Jacobs JP, Prager RL (2013). Report from the Society of Thoracic Surgeons National Database Workforce: clarifying the definition of operative mortality. World J Pediatr Congenit Heart Surg.

[13] Pierard S, de Meester C, Seldrum S (2014). Impact of preoperative symptoms on postoperative survival in severe aortic stenosis: implications for the timing of surgery. Ann Thorac Surg.

[14] McCarthy FH, Savino DC, Brown CR (2017). Cost and contribution margin of transcatheter versus surgical aortic valve replacement. J Thorac Cardiovasc Surg.

[15] Buchwald AB, Meyer T, Scholz K, Unterberg C, Schorn B (2001). Efficacy of balloon valvuloplasty in patients with critical aortic stenosis and cardiogenic shock--the role of shock duration. Clin Cardiol.

[16] Frerker C, Schewel J, Schluter M (2016). Emergency transcatheter aortic valve replacement in patients with cardiogenic shock due to acutely decompensated aortic stenosis. EuroIntervention.

[17] Faridi KF, Yeh RW, Poulin MF (2019). Treating symptomatic aortic stenosis with transcatheter aortic valve replacement: is there time to wait?. J Am Heart Assoc.

[18] Malaisrie SC, McDonald E, Kruse J (2014). Mortality while waiting for aortic valve replacement. Ann Thorac Surg.

